# Does bronchoalveolar lavage lymphocytosis predict prognosis in fibrotic hypersensitivity pneumonitis, and is this relationship influenced by low-dose immunomodulatory therapy at the time of BAL?

**DOI:** 10.3389/fmed.2026.1877266

**Published:** 2026-07-21

**Authors:** Punchalee Kaenmuang, Fiammetta Danzo, Simon Bax, Richard J. Hewitt, Maria Kokosi, Vasileios Kouranos, Felix Chua, Peter M. George, Gisli Jenkins, Athol U. Wells, Carmel J. W. Stock, Piersante Sestini, Elisabetta A. Renzoni

**Affiliations:** 1 Respiratory and Respiratory Critical Care Unit, Division of Internal Medicine, Faculty of Medicine, Prince of Songkla University, Songkhla, Thailand; 2 Interstitial Lung Disease Unit, Royal Brompton Hospital, Guy’s and St Thomas’ National Health Service Foundation Trust, London, United Kingdom; 3 Margaret Turner Warwick Centre for Fibrosing Lung Disease, National Heart and Lung Institute, Imperial College London, London, United Kingdom; 4 Department of Biomedical and Clinical Sciences (DIBIC), Università degli Studi di Milano, Milan, Italy; 5 King's Centre of Lung Health, School of Immunology and Microbial Sciences, Faculty of Life Sciences and Medicine, King's College London, London, United Kingdom; 6 Department of Medical and Surgical Sciences and Neurosciences, University of Siena, Siena, Italy

**Keywords:** bronchoalveolar lavage, bronchoalveolar lavage lymphocytosis, composite physiologic index, fibrotic hypersensitivity pneumonitis, pulmonary function test, survival

## Abstract

**Background:**

Bronchoalveolar lavage (BAL) lymphocytosis is associated with improved prognosis in fibrotic hypersensitivity pneumonitis (fHP), although its prognostic value is unclear in patients receiving immunomodulatory treatment. We investigated whether treatment tapered to the lowest tolerated level at the time of BAL modifies associations with disease severity, one-year lung function, and transplant-free survival.

**Methods:**

We analysed 247 fHP patients undergoing BAL, including 162 untreated and 85 receiving immunomodulatory therapy at the time of BAL. Associations between BAL lymphocytosis and baseline composite physiologic index (CPI), one-year CPI change, and transplant-free survival were assessed using multivariable flexible parametric survival models incorporating treatment status and interaction terms, adjusted for covariates.

**Results:**

Mean age was 63 years, 43% male, 37% ever-smokers. Treated patients at the time of BAL were younger, with more severe disease (higher CPI). Mean BAL lymphocyte percentages were similar between two groups and inversely associated with baseline CPI in both. Higher BAL lymphocyte% were associated with more favourable CPI change in both untreated (−1.7 points per 10% increase; 95%CI −2.5–−0.8, *p* < 0.0001) and treated patients at BAL (−2.0 points; 95% CI −3.1–−0.9, p < 0.0001), with no treatment-by-BAL lymphocyte interaction (*p* = 0.64). Higher BAL lymphocyte% independently predicted lower mortality both in untreated (HR 0.87 per 10% increase, *p* = 0.036) and treated patients at the time of BAL (HR 0.78, *p* = 0.025), with no treatment-by-BAL lymphocyte interaction (*p* = 0.39).

**Conclusion:**

BAL lymphocytosis was associated with less severe disease, favourable one-year CPI change and better survival, irrespective of background immunomodulatory therapy.

## Introduction

Fibrotic hypersensitivity pneumonitis (fHP) is characterised by a variable and often unpredictable clinical course. Bronchoalveolar lavage (BAL) lymphocytosis is not only a diagnostic hallmark of fHP but may also serve as an indicator of underlying inflammatory activity and disease behaviour. Several studies have reported that higher BAL lymphocyte percentages were associated with better survival outcomes in patients with fHP (1–3). However, only a small number of studies have examined the relationship between BAL lymphocytes and short-term lung function changes (4, 5). Furthermore, to our knowledge, there is no data to assess whether immunomodulatory treatment (corticosteroids and/or immunosuppressive drugs) at the time of BAL modifies the association between BAL lymphocytosis and outcomes.

Ideally, BAL is performed in patients who are not on immunomodulatory treatment, based on the unverified assumption that anti-inflammatory/ immunosuppressive treatment may alter baseline BAL lymphocyte levels and consequently affect their association with clinical outcomes. However, this assumption has not been proven, and existing studies that include patients on immunomodulatory treatment at the time of BAL do not provide separate analyses for this subgroup (6).

In real-world clinical practice, it may not be feasible to withhold immunomodulatory treatment at the time of BAL, as patients may have been empirically started on treatment prior to referral to an ILD specialist centre and may experience worsening symptoms on tapering corticosteroids or steroid-sparing agents. We therefore set out to examine whether immunomodulatory treatment, tapered to the lowest tolerated level at the time of BAL, influences the relationship between BAL lymphocytosis and clinical outcomes, including changes in lung function at 1 year and transplant-free survival.

## Materials and methods

Consecutive patients with fHP followed at the Royal Brompton Hospital (RBH) ILD Unit between 2006 and 2019 were considered. A diagnosis of fHP was made via multidisciplinary discussion, with fibrosis on CT defined by the presence of at least one of the following features: reticulation, traction bronchiectasis or bronchiolectasis, honeycombing (7, 8). Patients were included if they underwent BAL for diagnostic purposes within 3 months of baseline lung function tests. A total of 247 patients met inclusion criteria. Histological confirmation of fHP was available for 68 patients (27.5%). Clinical and treatment data at the time of BAL, as well as the treatment plan after BAL, were retrieved from clinical records. BAL was performed either in the absence of immunomodulatory treatment or while patients were receiving the lowest dose considered feasible by the managing physician (Supplementary Table S1). BAL was undertaken for diagnostic purposes irrespective of immunomodulatory treatment status at the time of BAL. The composite physiologic index (CPI) was used as a lung function-based marker of ILD severity (9). CPI was calculated using a previously validated formula incorporating percent predicted values of forced expiratory volume in 1 s (FEV_1_), forced vital capacity (FVC), and diffusing capacity for carbon monoxide (DLCO) as follows: CPI = 91.0 - (0.65 x % predicted DLCO) - (0.53 x % predicted FVC) + (0.34 x % predicted FEV_1_). All-cause mortality was collected until death, transplant, loss to follow-up or end of study period (31 May 2024). The study received ethical approval (REC: 13/LO/0857).

Our primary objective was to assess whether immunosuppressive treatment reduced to the lowest tolerated level at the time of BAL modifies the association between BAL lymphocytosis and: (i) contemporary disease severity (CPI at baseline), (ii) change in disease severity after 1 year (ΔCPI from baseline to 12 months), and (iii) overall survival. Accordingly, the primary estimand was the treatment-by-BAL lymphocyte percentage interaction term in multivariable linear regression models (for CPI and ΔCPI) and in survival models (for time-to-event outcomes). Immunomodulatory therapy (corticosteroids and/or immunosuppressive therapy) at the time of BAL will hereafter be referred to as “treatment.”

Secondary objectives were to evaluate the association between BAL lymphocyte percentage and baseline values, as well as 1-year changes of the individual CPI components (FVC, FEV_1_, and DLCO), all expressed as percent predicted. Furthermore, when no evidence of effect modification by treatment at the time of BAL was observed for a given endpoint, we also reported the overall association between BAL lymphocyte percentage and that endpoint, adjusted for treatment.

One-year changes in lung function parameters were evaluated in patients who underwent follow-up lung function tests (PFTs) at 1 year (±6 months) after BAL. Changes were calculated as the difference between the two measurements divided by the time interval between PFTs and expressed as annualized change in CPI or percent predicted lung function. Immunomodulatory treatment was initiated (in those not receiving treatment at the time of BAL) or intensified (in those receiving the lowest feasible dose at BAL) within 1 month of BAL (Supplementary Table S2) and was continued for at least 1 year in all patients, except for two who discontinued early due to side effects.

Linear regression models were used to evaluate the association between BAL lymphocyte percentage and baseline lung function, as well as one-year changes. Models included treatment at the time of BAL (dichotomous) and its interaction with BAL lymphocyte percentage, and were adjusted for sex, age and smoking history. An inciting antigen was identified in 112 of 247 patients (45.3%) but was not retained in final models as it was not significantly associated with any of the outcomes.

Possible non-linearity of continuous covariates was assessed using restricted cubic splines (3 degrees of freedom), testing the significance of their non-linear components using the Wald test. Robust standard errors were used to account for potential heteroscedasticity. Additional model assumptions were assessed using residual diagnostics. Model fit was assessed using Akaike and Bayesian information criteria (AIC and BIC).

Royston–Parmar restricted cubic spline flexible parametric survival models (10, 11) were used to evaluate associations with transplant-free survival over a period of 8 years from BAL, during which graphical inspection of Nelson–Aalen cumulative hazards did not suggest departures from proportionality. Corresponding Cox proportional hazards models were used to assess proportionality violations via scaled Schoenfeld residuals. When violations were detected, time-dependent interactions with increasing degrees of freedom were added. Final model specification was guided by the lowest AIC and BIC, and by comparing nested models using likelihood ratio tests. Hazard ratios for CPI score and BAL lymphocyte percentage are reported per 10-point increase. For graphical display, 68.3% CIs were used to approximate ±1 standard error.

In a sensitivity analysis, patients untreated at the time of BAL but previously exposed to immunosuppressive therapy were excluded.

A *p* value ≤0.05 (two-tailed) was used as a conventional threshold to flag potentially significant associations. All analyses were performed using Stata version 19 (StataCorp, College Station, TX).

## Results

At the time of BAL, there were 162 patients who had been untreated for at least 1 month, and 85 who were receiving treatment. Of the patients not receiving treatment at the time of BAL, 145 were completely treatment naive, whereas 17 had been treated before for a median of 99 (IQR 60–182) days but had been free of treatment for 166 (85–345) days before BAL, with none receiving treatment in the previous month, and only four in the previous 3 months. Patients receiving treatment at the time of BAL had been on corticosteroids for a median of 274 (174–706) days, with a starting dose of prednisone of 24 ± 11 mg (Mean ± SD), tapered to 12 ± 8 mg at the time of BAL. Eleven patients were also taking immunosuppressive drugs (further details in Supplementary Table S1). Anthropometric and clinical characteristics of the two groups are presented in Table 1. Patients on treatment at the time of BAL were on average younger, had lower lung volumes, and a slightly lower percentage of BAL eosinophils and mast cells, but no difference in BAL lymphocyte percentages was observed between the two groups.

**Table 1:** Anthropometric and clinical characteristics of the two groups.

Clinical characteristics	No treatment^*^ (*n* = 160)	On treatment^*^ (*n* = 87)	*p*-value
Age	64.5 ± 11.3	59.6 ± 10.8	0.001
Male sex	70 (43.8)	36 (41.4)	0.72
Ever smoker	63 (39.4)	29 (33.3)	0.35
Antigen identified	70 (43.8)	42 (48.3)	0.50
Lung function tests			
FVC % predicted	79.2 ± 23.1	70.8 ± 16.2	0.003
FEV_1_% predicted	79.2 ± 22.5	72.1 ± 17.3	0.01
DLCO % predicted	42.9 ± 12.6	40.7 ± 10.9	0.18
VA % predicted	65.7 ± 15.3	60.1 ± 12.8	0.004
KCO % predicted	75.3 ± 19.3	77.7 ± 18.1	0.34
CPI score	47.8 ± 11.7	51.5 ± 9.2	0.01
BAL cellularity			
Lymphocytes, %	27.6 ± 17.1	25.4 ± 16.2	0.33
Neutrophils, %	9.9 ± 10.1	11 ± 9.3	0.40
Eosinophils, %	5.1 ± 4.5	3.9 ± 3.7	0.04
Mast cells, %	0.6 ± 1	0.3 ± 0.6	0.03

*No or on treatment with immunomodulatory drugs at the time of BAL. Data are presented as number (percentage) for male sex, ever smoker, and antigen identified; all other data are presented as mean (Mean ± SD). BAL, bronchoalveolar lavage; CPI, composite physiologic index; DLCO, diffusing capacity for carbon monoxide; FEV_1_, forced expiratory volume in 1 s; FVC, forced vital capacity; KCO, carbon monoxide transfer coefficient; VA, alveolar volume.

### Correlation of % BAL lymphocytes and lung function

**Figure 1:**
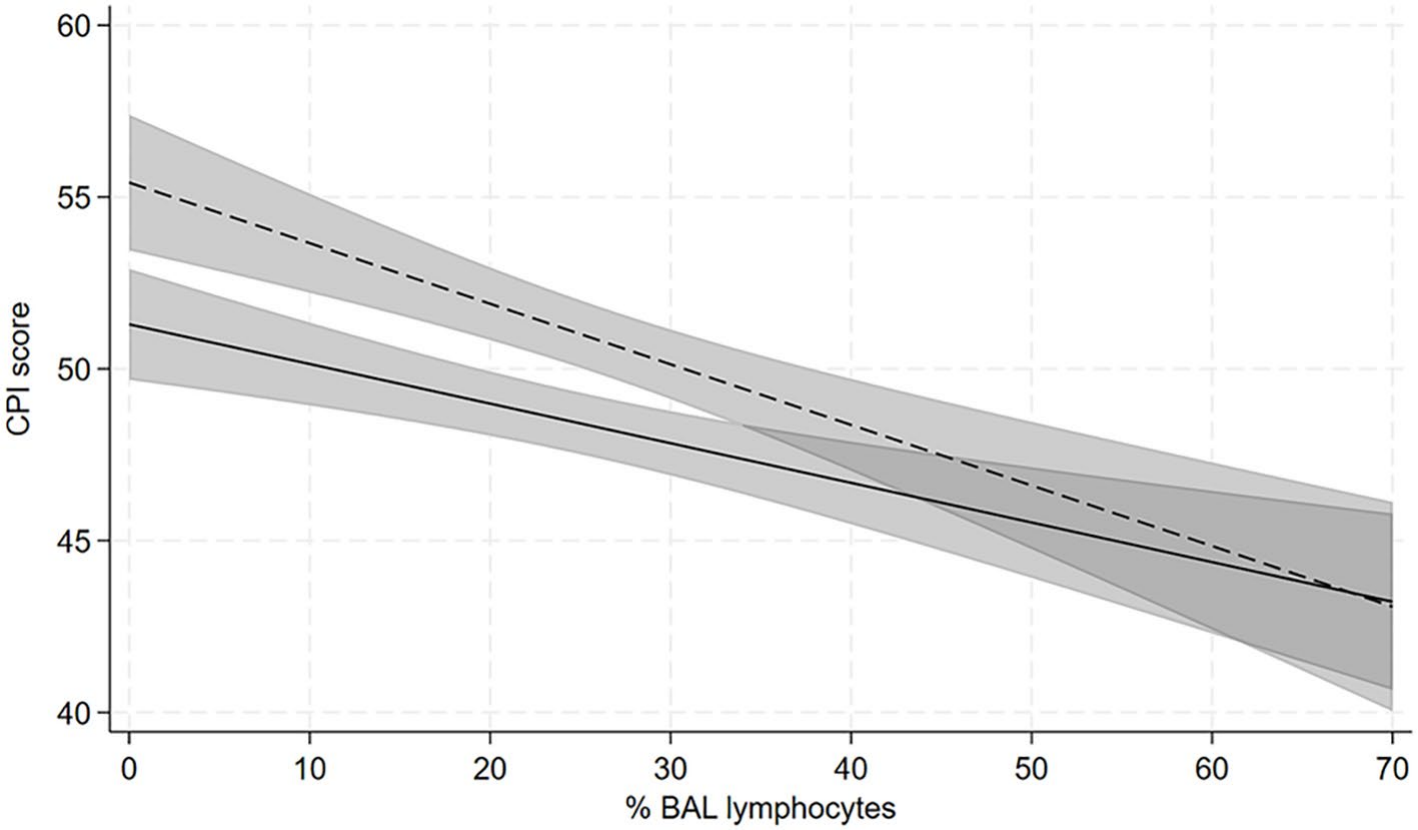
Adjusted regression of baseline CPI score versus % BAL lymphocytes in patients untreated (solid line) or on treatment (dashed line) at the time of BAL. Lines represent model-based predicted values from a linear regression including a treatment-by-lymphocyte interaction term. Shaded areas represent standard error bands; darker shading indicates overlap of the bands.

The mean BAL lymphocyte percentage did not differ significantly between treatment groups (Table 1), even after adjustment for sex, age, smoking history, and CPI score (*p* = 0.21). In a model adjusted for sex, age and smoking history, baseline CPI was inversely correlated with BAL lymphocyte percentage in both groups (Figure 1). In patients untreated at the time of BAL, CPI decreased by −1.2 (95%C.I. −2.2–−0.8) for every 10% increase in BAL lymphocytes (*p* = 0.04), and by −1.8 (95%C.I. −3.1–−0.5) in treated patients (*p* = 0.009) (Table 2). There was no evidence of interaction between treatment status at the time of BAL and BAL lymphocyte percentage (−0.6; 95%C.I. (−2.3–1.1), *p* = 0.49), indicating similar slopes across the two groups (Table 2). No departure from linearity was observed for BAL lymphocyte percentage or age.

**Table 2:** Associations between BAL lymphocyte percentage and concurrent lung function parameters, estimated from a linear regression model with a treatment–lymphocyte interaction, showing the effect of BAL lymphocytes separately in untreated and treated patients, together with the adjusted difference between treatment groups^†^.

Lung function parameters	Effect per 10% increase in BAL lymphocytes		Interaction
	Untreated	On-treatment	
CPI score	−1.2 (−2.2–−0.8)^0.04^	−1.8 (−3.1–−0.5)^0.009^	−0.6 (−2.3–1.1)^0.49^
FVC % predicted	3.5 (1.7–5.4)^<0.0001^	3.6 (1.5–5.7)^0.001^	0 (−1.4–1.5)^0.97^
FEV1% predicted	3.0 (1.2–4.8)^<0.0001^	3.3 (1.2–5.3)^0.002^	0.3 (−1.1–1.6)^0.85^
DLCO % predicted	0.6 (−0.5–1.8)^0.3^	1.4 (0–2.9)^0.05^	0.8 (−1.1–2.7)^0.4^

^†^Values are regression coefficients (β) with 95% confidence intervals; *p*-values are shown in superscript. In the first two columns, values represent the change in the parameter listed in each row per a 10% increase in BAL lymphocytes, shown separately for untreated and treated patients. *p*-values in these columns test whether the association within each group differs from zero. The column “Interaction” reports “the interaction term between treatment status and BAL lymphocyte percentage, testing whether the slopes differ significantly between the two groups (adjusted for sex, age and smoking history). The main effects of treatment at the time of BAL (i.e. the adjusted difference between treated and untreated patients, with all other covariates held constant) were not statistically significant for any outcome and are omitted for clarity. All models are adjusted for sex, age, and smoking status.

Secondary analyses of CPI components (FVC, FEV_1,_ and DLCO) confirmed consistent positive associations with BAL lymphocyte percentage in both groups, except for DLCO which was not significantly linked in untreated patients. Effect estimates were similar between the two groups (Table 2 and Supplementary Figure S1).

### Correlation of % BAL lymphocytes and one-year lung function changes

One-year CPI change data were available for 235 patients (mean interval 0.97 years, SD 0.02), with no difference between the two groups. Four patients (two in each group) died within 18 months after BAL without follow-up testing. Mean CPI change did not differ from zero and was similar in untreated and treated patients at the time of BAL [−1.1 (SD 8.6) vs. −1.2 (SD 9.5) CPI points/year, *p* = 0.99].

**Figure 2:**
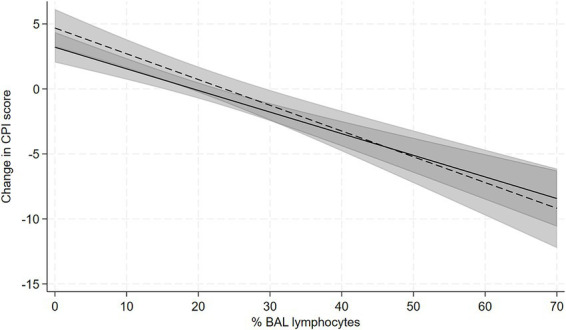
Adjusted regression of one-year changes in CPI score versus % BAL lymphocytes in patients untreated (solid line) or on treatment (dashed line) at the time of BAL. Lines represent model-based predicted values from a linear regression including a treatment-by-lymphocyte interaction term. Shaded areas represent standard error bands; darker shading indicates overlap of the bands.

In a linear regression model adjusted for sex, age, smoking and baseline CPI (Table 3), higher BAL lymphocyte percentage was associated with a greater reduction in CPI over 1 year. In patients untreated at the time of BAL, CPI decreased by 1.7 points per 10% increase in BAL lymphocytes (95% CI: −2.5– −0.8, *p* < 0.0001), and in treated patients by −2.0 points (95% CI: −3.1–−0.9, *p* < 0.0001) (Table 3). There was no evidence of non-linearity for BAL lymphocyte percentage or age. No significant interaction was observed between treatment status at the time of BAL and BAL lymphocyte percentage (interaction term −0.3, 95% CI −1.7**–**1.0, *p* = 0.64) (Table 3), and regression lines were similar between the two groups (Figure 2). Higher BAL lymphocyte percentages were associated with more favourable one-year CPI change. While low lymphocyte percentages were associated with one-year functional decline, higher percentages were associated with stability or improvement, with predicted trajectories crossing the null change line at approximately 20–30% BAL lymphocytes. Results were unchanged whether or not baseline CPI was adjusted for (Supplementary Table S3). Excluding treatment status at the time of BAL improved model fit (lower AIC/BIC) values, and in this model, a 10% increase in BAL lymphocytes was associated with a 1.8-point CPI reduction in the overall cohort (95% CI 1.1–2.5, *p* < 0.001).

**Table 3:** Associations between BAL lymphocyte percentage and one-year change in lung function parameters, estimated from a model with a treatment–lymphocyte interaction, showing the effect of BAL lymphocytes in patients untreated or treated at the time of BAL and the adjusted difference between treatment groups^†^.

Lung function parameters	Effect per 10% increase in BAL lymphocytes		Interaction
	Untreated	On-treatment	
CPI score	−1.7 (−2.5 –−0.8)^<0.0001^	−2.0 (−3.1–−0.9)^<0.0001^	−0.3 (−1.7–1.0)^0.64^
FVC % predicted	1.9 (0.8–3)^0.001^	1.6 (0.5–2.7)^0.005^	−0.3 (−1.8–1.2)^0.68^
FEV1% predicted	1.1 (0.04–2.1)^0.042^	1.7 (0.6–2.9)^0.003^	0.7 (−0.8–2.1)^0.37^
DLCO % predicted	1.4 (0.5–2.4)^0.002^	2.6 (1.3–3.9)^<0.0001^	1.1 (−0.4–2.7)^0.2^

†Values are regression coefficients (β) with 95% confidence intervals; *p*-values are shown in superscript. In the first two columns, values represent the change in the parameter listed in each row per a 10% increase in BAL lymphocytes, shown separately for untreated and treated patients. *p*-values in these columns test whether the association within each group differs from zero. The column “Interaction” reports the interaction term between treatment status and BAL lymphocyte percentage, testing whether the slopes differ significantly between the two groups (adjusted for sex, age and smoking history). The main effects of treatment at the time of BAL (i.e., the adjusted difference between treated and untreated patients, with all other covariates held constant) were not statistically significant for any outcome and are omitted for clarity. All models are adjusted for sex, age, smoking status, and baseline CPI.

Secondary analyses showed consistent associations across CPI components (FVC, FEV_1_, and DLCO), with no effect modification by treatment (Table 3 and Supplementary Figure S2).

### Association with survival

Eight-year survival did not differ between patients treated and untreated at the time of BAL in unadjusted analysis (HR 1.05, 95% CI 0.87–1.36; *p* = 0.81) or after adjustment for sex, age, smoking history, CPI score and % BAL lymphocytes (HR 1.14, 95% CI 0.93–1.38, *p* = 0.51) (Supplementary Figure S3).

A violation of the proportional hazards assumption for smoking history was addressed by including an interaction term with the natural logarithm of time. No non-linearity was detected for continuous covariates. There was no interaction between CPI and BAL lymphocyte percentage. Due to the linear associations with survival, CPI and BAL lymphocyte percentage were analysed as continuous variables.

In the multivariable model adjusted for age, sex, smoking history, and treatment status at the time of BAL, higher BAL lymphocyte percentage was associated with lower mortality (HR 0.84 per 10% increase in BAL lymphocyte percentage, 95% CI 0.76–0.94, *p* = 0.003), while higher CPI was associated with increased mortality (HR 1.62 per 10-point increase, 95% CI 1.32–1.99, *p* < 0.001).

The inverse association between BAL lymphocytes and survival remained significant both in untreated patients at the time of BAL (HR 0.87 per 10% increase, 95% CI 0.76–0.99, *p* = 0.036) and treated patients (HR 0.78, 95% CI 0.62–0.97, *p* = 0.025) even after inclusion of an interaction term, itself not significant (HR 0.89, 95% CI 0.69–1.15, *p* = 0.39). CPI estimates were unchanged (HR per 10-point increase 1.61, 95% CI 1.31–1.98, *p* < 0.001). Results were similar after excluding patients with prior immunosuppressive exposure but not receiving treatment at the time of BAL (data not shown).

**Figure 3:**
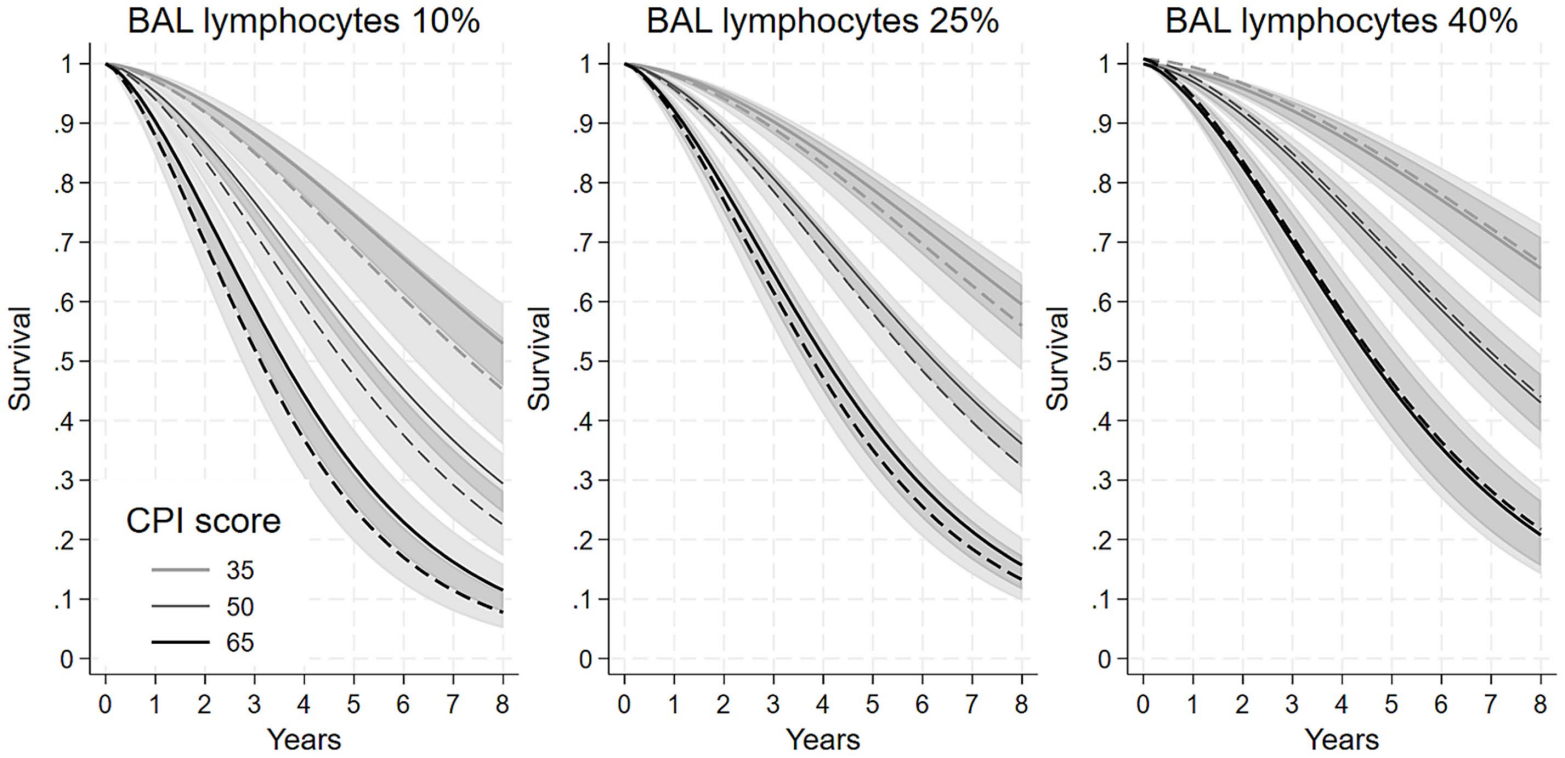
Model-predicted survival in patients untreated (solid lines) or receiving treatment (dashed lines) at the time of BAL, at three levels of BAL lymphocyte percentage (10, 25, and 40%; panels) and CPI score (35, 50, and 65; lines with increasing darkness), standardised for sex, age and smoking history. Shaded areas represent ±1 SE; darker shading indicates overlapping bands.

In the absence of an established cutoff for BAL lymphocyte percentage, model-predicted survival is illustrated in Figure 3 at selected equally spaced values of CPI (35, 50, and 65) and BAL lymphocyte percentage (10, 25, and 40%). These values were chosen to represent points across continuous scales of the two variables, and do not imply clinical cut-offs. Consistent with the absence of interaction, predicted survival was similar in treated and untreated patients across all combinations of CPI score and BAL lymphocyte percentage.

As treatment and its interaction with BAL lymphocytes did not improve model fit (Supplementary Table S4), a parsimonious model excluding these terms was retained (Supplementary Table S4). In this simpler model, baseline CPI remained a strong predictor of mortality, while BAL lymphocyte percentage showed an independent inverse association with mortality, with progressively lower risk across increasing lymphocyte levels (Supplementary Figure S4) across the range of CPI values. Although no interaction was detected between CPI and BAL lymphocytes, their opposite effects resulted in graded differences in predicted survival across their observed ranges. For all analyses, similar findings were obtained using Cox proportional hazards models.

## Discussion

In this study, BAL lymphocytosis was consistently associated with less severe disease at baseline, more favourable one-year lung function evolution, and improved survival in patients with fHP. Importantly, these associations were not significantly modified by immunomodulatory therapy administered at the lowest dose considered clinically tolerable at the time of BAL. These findings suggest that BAL lymphocytosis retains prognostic value even in patients receiving immunomodulatory treatment at the time of BAL, at least under the conditions of this study.

Our findings help address limited evidence on the impact of concurrent treatment on BAL lymphocytosis. Previous BAL-based studies have included substantial proportions of patients receiving corticosteroid or immunosuppressive therapy, although treatment timing relative to BAL is inconsistently reported (1, 6, 12, 13). In our cohort, 35% of patients were receiving immunomodulatory therapy at the time of BAL, suggesting that this scenario is common in clinical practice.

Although BAL has been extensively investigated in fHP and is incorporated into diagnostic guidelines (7, 8), data on the effects of corticosteroids or other immunosuppressants on BAL lymphocytes are scarce. In our cohort, BAL lymphocyte percentages did not differ significantly between untreated patients and those receiving relatively low-dose therapy at the time of BAL. While BAL lymphocytosis is widely regarded as a marker of immune activity in hypersensitivity pneumonitis (3, 6, 12), immunomodulatory therapies may exert predominantly functional or regulatory effects without reducing lymphocyte numbers (14, 15). Accordingly, the present findings should be interpreted as applying primarily to BAL lymphocyte percentages; treatment withdrawal may still be required when functional assays or immunophenotypic analyses of BAL lymphocytes are planned. Therapy was associated with modest but significant reductions in BAL eosinophils and mast cells, suggesting greater sensitivity of these cell types to treatment (16–18).

Several studies report greater short- to mid-term improvement in lung function following treatment initiation in patients with higher BAL lymphocyte counts (2, 5, 19). Although no consensus threshold defines “high” lymphocytosis, this observation has been replicated using cut-offs ranging from 20 to 40% (2, 5, 19). In this study, we analysed BAL lymphocyte percentage as a continuous variable, finding no evidence of non-linearity. Similarly, Dhooria et al. reported a graded effect across successive 10-percentage-point increases of BAL lymphocytes (4).

BAL lymphocyte percentage appeared to stratify patients into two functional trajectories: lower lymphocyte proportions were associated with one-year decline, whereas higher proportions were associated with stability or improvement. Predicted trajectories crossed the null-change line at approximately 20–30% BAL lymphocytes, suggesting a transition zone rather than a discrete threshold. We observed no difference in the association between BAL lymphocyte percentage and changes in CPI or other respiratory function parameters according to treatment status at BAL. All patients received relatively high-dose immunomodulatory therapy between the two lung function assessments. However, the absence of an untreated comparator and the retrospective nature preclude conclusions regarding treatment effectiveness.

De Sadeleer et al. (5) reported that lung function improvement was largely confined to patients without honeycombing on HRCT, although subsequent annual FVC decline did not differ. While we did not include HRCT data, we used CPI as a surrogate marker of disease severity (20). The association between BAL lymphocytes and improved one-year lung function change remained significant even after adjustment for baseline CPI, arguing against confounding by baseline disease severity.

Patients with fHP may show mixed fibrotic and non-fibrotic radiological changes (21). BAL lymphocytosis has been negatively correlated with fibrotic extent in fibrotic ILDs (19, 22) and positively associated with features such as centrilobular nodules in fHP (23, 24). Taken together, these findings support the hypothesis that BAL lymphocytosis identifies patients with a lower propensity towards fibrotic progression. Nevertheless, we did not evaluate baseline and follow up HRCT patterns and extent of fibrosis, as this was beyond the scope of the present study. The potential interaction between radiologic features, including fibrosis extent, and BAL lymphocyte percentage remains to be clarified in future studies.

BAL lymphocyte percentage was also independently associated with improved survival, with no evidence of modification by treatment status at the time of BAL. These findings support the concept that BAL lymphocytosis may identify a biologically distinct fILD phenotype with a more favourable natural history and potential treatment responsiveness, although no conclusion with regards to response to treatment can be drawn from this study.

De Sadeleer et al. reported that patients with fHP and BAL lymphocytosis >20% showed better initial FVC change (5). Similarly, Cano-Jiménez et al. found that BAL lymphocytosis was associated with reduced progression and improved survival (1). Hill et al. (23) also reported an association between BAL lymphocytes and improved survival.

We did not attempt to define a threshold for “high” or “low” lymphocyte counts against survival. Although such thresholds may aid clinical risk stratification, they are unlikely to be captured by a single cut-off and will require integration with other factors, including radiology, lung function, demographic factors, comorbidities and biomarkers (5, 24).

Although BAL lymphocytosis was associated with less severe disease, it remained associated with better outcomes even in more advanced disease. CPI was the strongest predictor of mortality, with an HR of approximately 1.6 per 10-point increase, highlighting its prognostic value (25). In multivariable models, both lower CPI and higher BAL lymphocytes were independently associated with better survival, supporting their complementary use in clinical risk stratification.

This study has limitations. Patients receiving immunomodulatory therapy at the time of BAL had more severe disease, introducing potential selection bias. However, the absence of interaction between treatment status at the time of BAL and the associations between BAL lymphocytosis and multiple outcomes supports the robustness of our findings. Immunomodulatory therapy was reduced to the lowest tolerated dose prior to BAL, and we cannot determine whether higher doses would alter associations. Variability in treatment tapering may have influenced BAL cellular profiles. At the time of BAL, most treated patients were receiving corticosteroids, with only a minority on non-steroidal immunosuppressive agents, limiting conclusions about non-steroidal agents.

Although treatment was initiated or intensified after BAL in all patients and maintained for at least 1 year, its intensity varied. Without a matched untreated group, improvements in lung function and survival cannot be attributed to a treatment response. We also cannot comment on anti-fibrotic therapy, as these agents were not available for non-idiopathic pulmonary fibrosis at the time.

The group on no treatment at the time of BAL included 17 previously treated patients, which may have confounded comparisons. However, a sensitivity analysis excluding these patients showed similar results.

Given the retrospective design, no formal power calculation was performed. Confidence intervals were wide, suggesting limited precision. However, interaction estimates were consistently in the opposite direction to that expected if treatment at the time of BAL attenuated associations, arguing against a meaningful modifying effect.

Glucocorticoids are known to modulate lymphocyte activation and distribution, without necessarily inducing depletion (14, 15). They may therefore affect immune function rather than lymphocyte cell counts. The biological plausibility that corticosteroids may affect BAL lymphocyte percentages in fHP could derive, at least in part, from observations in pulmonary sarcoidosis, where this effect has been repeatedly documented (26–30). Much less evidence is available in pulmonary fibrosis, particularly in fHP. Historical serial BAL studies in pulmonary fibrosis did not demonstrate a consistent reduction in BAL lymphocyte percentages following corticosteroid treatment, even when relatively high prednisolone doses were used (31). These studies should be interpreted cautiously, as they predated current diagnostic classifications and likely included a heterogeneous mixture of fibrotic lung diseases (32). Nevertheless, they included a few patients with substantial BAL lymphocytosis who, by current criteria, might in some cases have been classified as fHP. Thus, while available data do not exclude an effect of higher prednisolone doses on BAL lymphocyte counts in fHP, direct evidence supporting such an effect remains limited. Accordingly, our findings should primarily be interpreted as applying to patients receiving maintenance immunomodulatory therapy reduced to the lowest tolerated dose at the time of BAL, although we cannot exclude the possibility that similar findings might also be observed at higher prednisolone doses. While discontinuation before BAL is preferable, dose minimisation may be a more practical option in more severe or rapidly progressive fHP. In this setting, BAL lymphocytosis appears to retain its prognostic value.

In conclusion, BAL lymphocytosis is a clinically meaningful biomarker in fHP. It is associated with less severe disease, more favourable early lung function trajectory, and improved survival, independent of disease severity. These associations appear unaffected by low-dose immunomodulatory therapy, mitigating concerns that such treatment may limit the interpretability of BAL findings.

## Data Availability

The datasets presented in this article are not readily available because ethical restrictions. Requests to access the datasets should be directed to Punchalee Kaenmuang, punchalee.k@psu.ac.th.
