# Supplementary material for: Does bronchoalveolar lavage lymphocytosis predict prognosis in fibrotic hypersensitivity pneumonitis, and is this relationship influenced by low-dose immunomodulatory therapy at the time of BAL?
Source: Front Med (Lausanne). 2026 Jul 21;13:1877266. doi: 10.3389/fmed.2026.1877266 (PMC13433517; doi:10.3389/fmed.2026.1877266)
Supplement: Supplementary file 1 [file Data_sheet_1.docx]

Supplementary Material

**Table S1. Baseline treatment data at the time of BAL**

| **Treatment data** | **n=247** |
| --- | --- |
| **Patients on no immunomodulatory treatment at time of BAL** | 162 (65.6) |
| Treatment-naive | 145 (58.7) |
| No treatment for at least 10 weeks | 17 (6.9) |
| **Patients on treatment at time of BAL** | 85 (34.4) |
| Prednisolone alone | 74 (28.3) |
| Immunosuppressive agents* | 11 (5.3) |
| - azathioprine | 8 (3.2) |
| - methotrexate | 2 (0.8) |
| - cyclophosphamide | 1 (0.4) |

^*^Immunosuppressive agents with or without background corticosteroids. Doses of immunosuppressive agents were the following: azathioprine daily (one patient on 25 mg, two 75 mg, two 100 mg and three 150 mg/day), two patients on weekly methotrexate (2.5 and 25 mg) and one patient on cyclophosphamide 100 mg daily.

**Table S2. Treatment initiated or increased after bronchoalveolar lavage**

| **Treatment data** | **n = 247** |
| --- | --- |
| Corticosteroids only^†^ | 119 (48.2) |
| Corticosteroids and immunosuppressive agents^†, *^ | 104 (42.1) |
| Immunosuppressive agents only^‡^ | 24 (9.7) |

In all patients, immunomodulatory treatment was initiated or increased within one month of BAL

^†^While mean corticosteroid dose could not be calculated due to a proportion of patients being initially treated with high dose IV methylprednisolone, all patients received prednisolone (or prednisolone equivalent) at a dose ≥ 20 mg daily.

^*^Immunosuppressive agents used included mycophenolate (42 patients), azathioprine (45 patients), i.v. cyclophosphamide (12 patients), hydroxychloroquine (4 patients), rituximab (1 patient).

^‡^Immunosuppressive agents used included mycophenolate (8 patients), azathioprine (7 patients), i.v. cyclophosphamide (5 patients), hydroxychloroquine (3 patients), rituximab (1 patient).

**Table S3 Association between BAL lymphocyte percentage and one-year change in CPI score, shown with and without adjustment for baseline CPI†**

|  | **Unadjusted for baseline CPI** | **Adjusted for baseline CPI** |
| --- | --- | --- |
| **BAL lymphocytes (10%)** | -1.5 (-2.4–-0.7)*^<0.001^* | -1.7 (-2.5–-0.8)*^<0.001^* |
| **Baseline CPI** | - | -0.12 (-0.24–-0.01)*^0.04^* |
| **Treatment** | 0.98 (-2.57–4.54)*^0.59^* | 1.48 (-2.09–5.06)*^0.42^* |
| **Treatment x lymphocytes interaction** | -0.24 (-1.60–1.12)*^0.73^* | -0.32 (-1.65–1.02)*^0.64^* |
| **R^2^** | 0.14 | 0.16 |
| **AIC** | 1551.15 | 1547.81 |
| **BIC** | 1574.85 | 1574.89 |

Abbreviations: AIC, Akaike information criterion; BAL, bronchoalveolar lavage; BIC, Bayesian information criterion, CPI, composite physiological index

^†^The coefficient for BAL lymphocytes represents the change in annualised CPI per 10% increase in BAL lymphocytes in patients untreated at the time of BAL. The treatment coefficient represents the difference in annualised CPI change between treated and untreated patients. The interaction term represents how this association differs between these groups. Values are regression coefficients (β) with 95% confidence intervals; *P*-values are shown in superscript. Both models are adjusted for sex, age and smoking history. R² indicates the coefficient of determination.

**Table S4 Comparison of survival models including treatment at the time of BAL as an interaction with BAL lymphocytes, as a main effect only, or not included (95% CI)^†^**

| **Variable** | **Full model** | **Main effect model** | **Parsimonious model** |
| --- | --- | --- | --- |
| CPI (x 10) | 1.61 (1.31–1.98)*^<0.001^* | 1.62 (1.32–1.99)*^<0.001^* | 1.64 (1.33–2.01)*^<0.001^* |
| % BAL lymphocytes (x 10) | 0.87 (0.76–0.99)^‡^*^0.036^* | 0.84 (0.76–0.94)*^0.003^* | 0.84 (0.75–0.94)*^0.002^* |
| Treatment | 1.47 (0.73–2.95)*^0.277^* | 1.13 (0.77–1.68)*^0.513^* | - |
| Treatment × BAL lymphocytes | 0.89 (0.69–1.15)*^0.391^* | - | - |
| **Model fit** |  | | |
| AIC | 723.3252 | 722.0733 | 720.4974 |
| BIC | 758.0899 | 753.3615 | 748.3091 |
| Likelihood ratio test | -- | χ^2^=0.75, *P=0.387* | χ^2^=1.17, *P*=*0.557* |

Abbreviations: AIC, Akaike information criterion; BAL, bronchoalveolar lavage; BIC, Bayesian information criterion, CPI, composite physiological index

^†^Values are hazard ratios (HR) with 95% confidence intervals; *p*-values are shown in superscript. HRs for BAL lymphocytes and CPI are expressed per 10-unit increase. HRs for BAL lymphocyte percentage represent the effect in untreated patients (reference group); the interaction term captures the additional effect in treated patients. Treatment represents the effect of being on treatment at the time of BAL, at the reference value of BAL lymphocytes.
All models are adjusted for sex, age, and smoking history (coefficients not shown). The full model includes treatment, BAL lymphocytes, and their interaction. The main effect model includes treatment without the interaction term. The parsimonious model excludes both treatment and the interaction. Likelihood ratio tests compare each reduced model with the full model.

^‡^The HR for BAL lymphocytes in patients on treatment, obtained by linear combination of model coefficients, was 0.78 (0.62–0.97), *p* = 0.025.

**Figure S1** Adjusted regression models of baseline lung function parameters (FVC, FEV_1_, and DLCO) versus % BAL lymphocytes in untreated (solid line) and treated (dashed line) patients at the time of BAL. Lines represent model-based predicted values from linear regression models including a treatment-by-lymphocyte interaction term. Shaded areas represent standard error bands; darker shading indicates overlap of the bands.

**Figure S2** Adjusted regression models of one-year changes in lung function parameters (FVC, FEV_1_, and DL_CO_) versus % BAL lymphocytes in untreated (solid line) and treated (dashed line) patients at the time of BAL. Lines represent model-based predicted values from linear regression models including a treatment-by-lymphocyte interaction term. Shaded areas represent standard error bands; darker shading indicates overlap of the bands.

**Figure S3** Kaplan Meier plot of survival in untreated patients (solid line) or patients treated with immunomodulation (dashed line) at the time of BAL.

**Figure S4** Model-predicted survival at three levels of BAL lymphocyte percentage (10%, 25% and 40%; panels) and CPI score (35, 50 and 65; coloured lines), standardised for sex, age and smoking history. Shaded areas represent ±1 SE; darker shading indicates overlapping bands. Colours follow the viridis palette for perceptual uniformity and colour-vision accessibility.
